# Insights into Nitrogen-Associated Protein 50 (NAP50) as a Tyrosyl–DNA Phosphodiesterase in Dinoflagellates

**DOI:** 10.3390/microorganisms12112286

**Published:** 2024-11-11

**Authors:** Lu Tang, Nora Fung-Yee Tam, Winnie Lam, Thomas Chun-Hung Lee, Steven Jing-Liang Xu, Fred Wang-Fat Lee

**Affiliations:** 1 School of Science and Technology, Hong Kong Metropolitan University, Hong Kong SAR, China; tanglu17@mails.ucas.ac.cn (L.T.); fytam@hkmu.edu.hk (N.F.-Y.T.); lamw@hkmu.edu.hk (W.L.); chhlee@hkmu.edu.hk (T.C.-H.L.); sjlxu@hkmu.edu.hk (S.J.-L.X.); 2 State Key Laboratory of Marine Pollution, City University of Hong Kong, Hong Kong SAR, China

**Keywords:** dinoflagellate, nitrogen-associated protein 50 (NAP50), plastid protein, tyrosyl–DNA phosphodiesterase

## Abstract

Nitrogen-associated protein 50 (NAP50) is an abundant plastid protein with an unknown function identified in *Alexandrium affine* (Dinophyceae). No progress has been made in discovering the function of NAP50 since its first characterization in 2009. The present study is a continuation of work on the predicted function of NAP50. The results show that the NAP50 gene lacks introns but contains abundant base substitutions, consistent with the characteristics of dinoflagellate nuclear genes. The NAP50 protein is found to be widely expressed in dinoflagellate lineages through bioinformatics analysis and Western blotting, suggesting that NAP50 is not exclusive to *Alexandrium*, which differs from previous understandings. Phylogenetic analysis reveals that NAP50 belongs to the tyrosyl–DNA phosphodiesterase (TDP) family; however, it is structurally distinct from the TDP2 that is present in some dinoflagellate species. The three-dimensional structure and biological functions of NAP50 are predicted using deep learning algorithms. Based on evolutionary relationships and functional predictions, NAP50 may play a role in repairing plastid DNA damage and potentially contribute to the transcription of plastid genes in dinoflagellates.

## 1. Introduction

Dinoflagellates are a species-rich group of marine plankton that play a crucial role in oceanic primary production and biogeochemical cycles [1,2]. The symbiodiniacean zooxanthellae are important symbionts in the coral reef ecosystem [3], while certain free-living dinoflagellate species can grow rapidly under suitable conditions, leading to “red tides” [4]. The genetic information of dinoflagellates, particularly free-living ones, is largely unknown on a genome scale, mainly due to their large genome size (7–200 Gbp) [5] and atypical genome structure [6]. Current data show that dinoflagellate genomes encode a large proportion of “dark” proteins, that is, they have no significant sequence similarity to any proteins with a known function in databases [7]. The homologs of these dark proteins are highly divergent in distantly related dinoflagellate lineages, indicative of lineage-specific functional innovations [8]. Dark proteins represent a vast unknown area in the functional landscape of dinoflagellates. Studies on dark proteins provide valuable insights into the adaptations of dinoflagellates to their environments and evolutionary strategies employed by different dinoflagellate lineages.

Dinoflagellate plastids have a complex evolutionary history due to multiple endosymbiotic events, and exhibit intriguing characteristics such as the plasmid-like minicircles found in peridinin dinoflagellate plastids [9]. Nitrogen-associated protein 50 (NAP50) was first identified in *Alexandrium affine*, a harmful algal bloom-forming dinoflagellate species, in 2009. This nuclear-encoded plastid-targeted protein’s expression decreases more than 15-fold under nitrogen depletion conditions, which is likely regulated at the post-transcriptional level [10]. The breakdown of the NAP50 protein may contribute to nitrogen cycling, thereby sustaining algal survival under nitrogen deficiency [10]. NAP50 is abundantly expressed in the plastid of dinoflagellate *A. affine* [10], suggesting that NAP50 may play important biological roles in plastid function. However, due to the evolutionary uniqueness and diversity of dinoflagellate genomes, the function of the NAP50 protein still remains poorly characterized. NAP50 has received little follow-up research since its discovery despite its potential functions and abundant expression in plastids. Given the recent decipherment of dinoflagellate genomes, it is worthwhile to investigate the previously considered dark proteins such as NAP50 in different dinoflagellate lineages. This study aims to (i) investigate the conservation of NAP50 within dinoflagellate genomes; (ii) explore its distribution through Western blotting in different dinoflagellate lineages, for which genomic data are lacking; and (iii) employ deep learning-based methods to predict the structure and function of NAP50. This study reveals the genetic structure and phylogeny of NAP50, as well as its distribution in dinoflagellates and structure-based functional predictions. The findings of this study enhance our understanding of this dark protein (NAP50) that is abundantly expressed in the plastid, providing insights into the function of NAP50 in different dinoflagellate lineages.

## 2. Materials and Methods

### 2.1. Algal Strains and Cultivation

The dinoflagellates Alexandrium affine (CCMP 112), Alexandrium fundyense (CCMP1719), Alexandrium tamarense (Group IV) (CCMP1598), Amphidinium carterae (CCMP 121), Scrippsiella acuminata (CCMP 3239), and Prorocentrum hoffmannianum (CCMP 2804) were purchased from the National Center for Marine Algae and Microbiota (NCMA). Karenia mikimotoi (strain NIES2411) was sourced from the National Institute for Environmental Studies collection, Japan. Gymnodinium sp. was obtained from the Metropolitan Algal Repository and Supply, Hong Kong. All algae were grown in an L1-Si medium with a salinity of 30 ppt, and maintained at 22 °C with illumination at 50 μE m^−2^ s^−1^ under a 12:12 h light–dark cycle.

### 2.2. NAP50 Gene Structure

The DNA of *A. affine* was extracted from 100 mL of cells in the mid-log phase using the DNeasy Plant Pro Kit (Qiagen, Valencia, CA, USA). The potential exons of the NAP50 gene were confirmed through PCR, with primers designed based on its cDNA sequences (GQ480795.1) using Primer Premier 5 (Forward, CCGTGCCGTGTCTATTCT; Reverse, CCTTCTCGTATTCTGCGTTC). The PCR product was purified using the GeneJET Gel Extraction Kit (Thermo Fisher Scientific, Waltham, MA, USA) and sequenced by Tech Dragon Ltd. (Hong Kong, China).

### 2.3. Phylogenetic Analysis

The NAP50 protein sequence (ACV60537.1) was utilized to search similar sequences using BLAST from the National Center for Biotechnology Information (NCBI) database (https://www.ncbi.nlm.nih.gov/, accessed on 12 December 2023) and the Symbiodiniaceae and Alagl Genomic Resource (SAGER) database (http://sampgr.org.cn/, accessed on 12 December 2023). The top five hits with the lowest E values from different algal species in the NCBI database were downloaded, two of which were annotated as the TDP2 protein (CAE7230169.1 and CAE7810483.1), while the other three were annotated as “unnamed protein products” (CAE8638858.1, CAJ1431316.1, and CAI3974620.1). TDP2 from different algal species, including *Chlorella desiccata* (KAH7616892.1), *Gracilariopsis chorda* (PXF41120.1), *Seminavis robusta* (CAB9528062.1), *Symbiodinium natans* (CAE7551941.1), and *Symbiodinium microadriaticum* (OLQ15685.1), was sourced from the NCBI protein database. TDP2 sequences from animals, including *Homo sapiens* (NP_057698.2), *Bos taurus* (NP_001098811.1), *Gallus gallus* (NP_001383838.1), *Xenopus tropicalis* (NP_001016944.1), and *Danio rerio* (NP_001073171.1), as well as plants, such as *Arabidopsis lyrata* subsp. *lyrata* (XP_002892657.1) and *Brachypodium distachyon* (XP_003565702.1), were retrieved. Additionally, the TDP2 of *S. microadriaticum* (OLQ15685.1) was used to search its homologs within the genomes from the order Suessiales, resulting in three additional “unknown protein products” identified from *C. goreaui*, *E. voratum*, and *P. glacialis* (CAI3988574.1, CAJ1394444.1, and CAK0789787.1). The TDP family of proteins from the ciliate *Tetrahymena thermophila* SB210 (XP_001023542.1) was also downloaded. Multiple sequence alignments were conducted between NAP50, its similarity search hits from the NCBI database, and all known TDP2 protein sequences, using ClustalW in BioEdit software (version 7.7.1). All algal and ciliate sequences were aligned, and a phylogenetic tree was conducted using the neighbor-joining (NJ) method with a bootstrap value of 1000 in Mega 11.0 software.

### 2.4. Western Blotting

Western blotting was employed to investigate the distribution of NAP50 among dinoflagellate lineages. A total of 100 mL of each of the algal cultures was harvested for total protein extraction using the Trizol reagent (Invitrogen, Carlsbad, CA, USA) [10]. Total protein was separated by SDS-PAGE and transferred to a 0.45 μm thick nitrocellulose membrane. A polyclonal rabbit antibody against NAP50 was produced by Abiotech (Jinan, China). The membrane was blocked with 5% BSA and subsequently incubated with 1 μg/mL of the primary antibody at 4 °C overnight. After washing with a TBST buffer containing 1% BSA, the membrane was incubated with diluted HRP-conjugated goat anti-rabbit secondary antibody (30 ng/mL) (Thermo Fisher Scientific, Waltham, MA, USA). Non-bound conjugates were removed, and the membrane was developed using a SuperSignal^®^ West Pico Chemiluminescent Substrate Kit (Thermo Fisher Scientific, Waltham, MA, USA), following the manufacturer’s instructions. The imaging was performed using the ChemiDoc MP Imaging System (Bio-Rad, Hercules, CA, USA).

### 2.5. NAP50 Protein Structure and Function Prediction

The NAP50 protein sequence was subsequently submitted to D-I-TASSER (https://zhanggroup.org/D-I-TASSER/, (accessed on 12 December 2023) for protein structure and function prediction [11]. A large-scale metagenomic database, AlphaFold2 distances, and an automatic domain partition/assembly were utilized in the analysis. Protein function was predicted with Gene Ontology (GO) terms based on its confident structure model.

## 3. Results

### 3.1. NAP50 Is a Novel Dinoflagellate Protein Similar to TDP Family of Proteins

The present study found abundant base substitutions but no introns in the NAP50 gene (Supplementary Figure S1). NAP50 gene fragments (1126 bp) obtained through PCR using both genomic DNA and cDNA as templates exhibited a similarity of 90.4%. Out of 108 substitutions, 83 were synonymous, while the remaining 25 non-synonymous substitutions led to 19 amino acid changes (Supplementary Figure S1).

**Figure 1:**
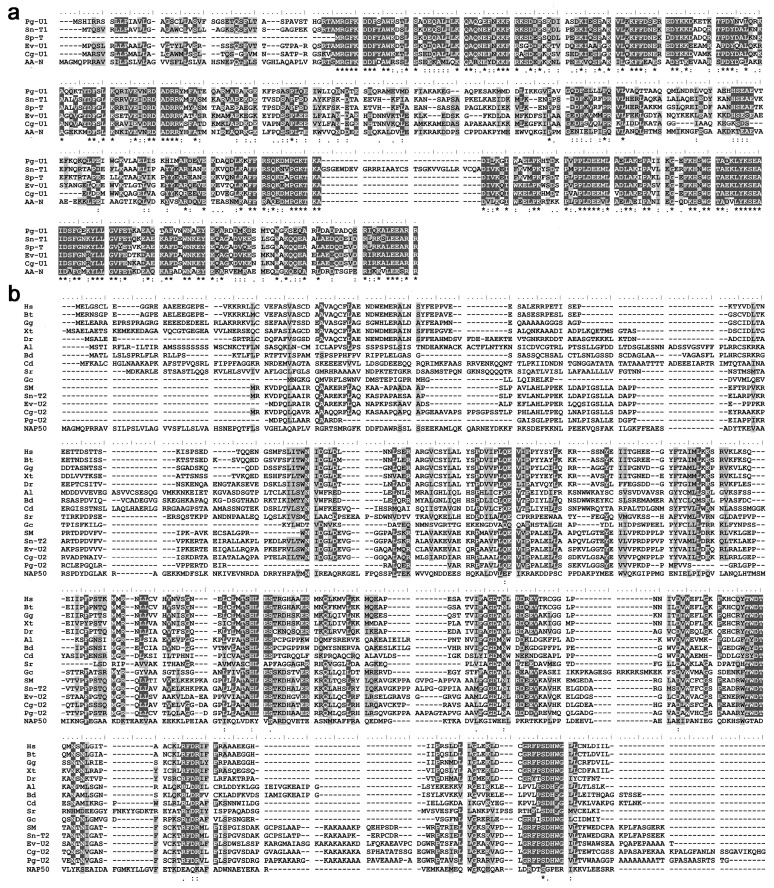
Analysis of multiple protein sequence alignment. Alignment of NAP50 with its homologs in Suessiales (**a**), and with TDP2 sequences from various species (**b**). Identical bases are marked with *, and similar bases are marked with . and :. Bases that share more than 60% identity or similarity at the same site are highlighted with black and gray shading, respectively. Abbreviations: Pg, *P. glacialis*; Sn, *S. natans*; Sp, *Symbiodinium* sp.; Ev, *E. voratum*; Cg, *C. goreaui*; AA, *A. affine*; Hs, *H. sapiens*; Bs, *B. taurus*; Gg, *G. gallus*; Xt, *X. tropicalis*; Dr, *D. rerio*; Al, *A. lyrata*; Bd, *B. distachyon*; Cd, *C. desiccata*; Gc, *G. chorda*; Sr, *S. robusta*; Sm, *S. microadriaticum*. U, uncharacterized named product; T, TDP2; N, NAP50. Sequences with the same annotation in the NCBI protein database were distinguished by adding a number at the end of the names.

**Figure 2:**
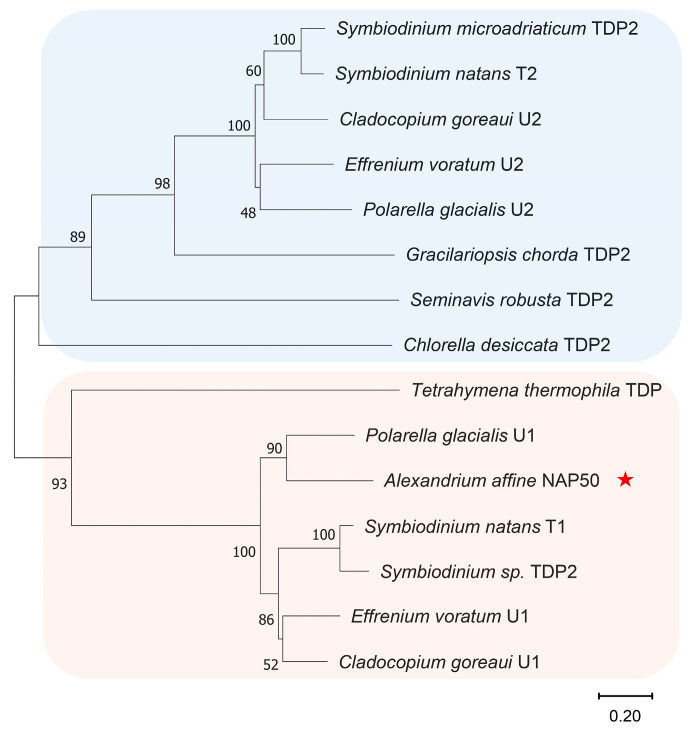
Phylogenetic analysis. Neighbor-joining (NJ) tree showing the phylogenetic relationships between NAP50 and TDP, with bootstrap support values displayed on the branches. The two divergent clades are highlighted in different colors, in which the initially identified NAP50 is marked with an asterisk. Abbreviations: Pg, *P. glacialis*; Sn, *S. natans*; Sp, *Symbiodinium* sp.; Ev, *E. voratum*; Cg, *C. goreaui*; AA, *A. affine*; Hs, *H. sapiens*; Bs, *B. taurus*; Gg, *G. gallus*; Xt, *X. tropicalis*; Dr, *D. rerio*; Al, *A. lyrata*; Bd, *B. distachyon*; Cd, *C. desiccata*; Gc, *G. chorda*; Sr, *S. robusta*; Sm, *S. microadriaticum*. U, uncharacterized named product; T, TDP2; N, NAP50. Sequences with the same annotation in the NCBI protein database were distinguished by adding a number at the end of the names.

Several sequences from the genus *Alexandrium* were found to have high similarities (68–78%) to the NAP50 protein sequence using the Blast search in the SAGER database (Supplementary Figure S2). Five protein sequences with similarities of 41–55% in the dinoflagellate order Suessiales were also identified from the NCBI database (Figure 1a). Although two of these five sequences were annotated as TDP2, the NAP50 sequence exhibited notable differences in conserved sites to the TDP2 sequences of animals, plants, and algae (Figure 1b). NAP50 proteins and typical TDP2 proteins were both identified in the genomes of the four dinoflagellates, that is, *P. glacialis*, *E. voratum*, *C. goreaui,* and *S. natans*; however, only the TDP2 protein was identified in *S. microadriaticum*, and only the NAP50 protein was found in *Symbiodinium* sp. (CCMP2592). Further phylogenetic analysis revealed that these NAP50 sequences clustered into a distinct branch with typical TDP2 proteins and were more closely related to the TDP family of proteins in ciliates (Figure 2).

### 3.2. Distribution of NAP50 in Dinoflagellates Using Western Blotting

**Figure 3:**
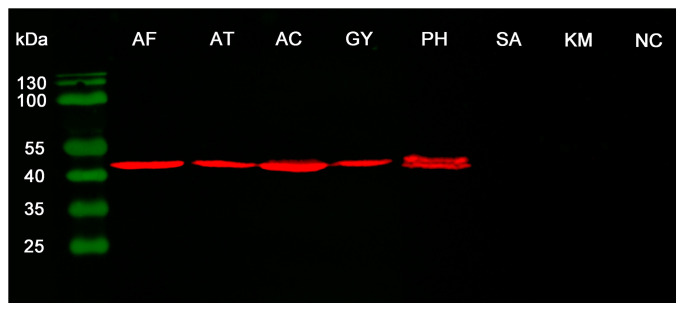
Detection of NAP50 distribution in dinoflagellates using Western blotting. The ladders are detected with a colorimetric method in the green channel, and the target proteins are detected with chemiluminescence in the red channel. AF, *A. fundyense*; AT, *A. tamarense*; AC, *A. carterae*; GY, *Gymnodinium* sp.; PH, *P. hoffmannianum*; SA, *S. acuminata*; KM, *K. mikimotoi*; NC, negative control.

Due to the limited dinoflagellate genome data, Western blotting was employed in a broader range of dinoflagellate species. The results showed that NAP50 was widely distributed in dinoflagellates, including species from the genera *Alexandrium* (*A. affine* and *A. fundyense*), *Amphidinium* (*A. carterae*), *Gymnodinium*, and *Prorocentrum* (*P. hoffmannianum*) (Figure 3). Two bands with very similar molecular weights near 50 kDa were found in *P. hoffmannianum*. The NAP50 protein was not detected in all the tested dinoflagellate species, including *S. acuminata* and *K. mikimotoi*.

### 3.3. The Predicted NAP50 Protein Structure and Function

**Figure 4:**
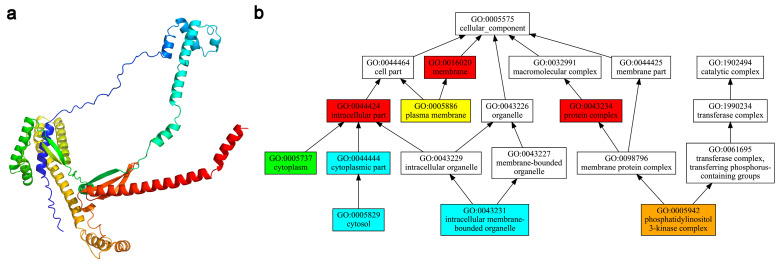
The predicted NAP50 structure and function. (**a**) NAP50 structure is modeled using the D-I-TASSER algorithms; (**b**) Gene Ontology (GO) terms in the cellular component category annotated for the NAP50 function. Terms are highlighted with different colors based on their confidence scores: red for 0.9–1.0, orange for 0.8–0.9, yellow for 0.7–0.8, green for 0.6–0.7, and blue for 0.5–0.6.

The structure of the NAP50 protein was successfully folded with an estimated TM score of 0.81 using D-I-TASSER (Figure 4a). The functions of the NAP50 protein were predicted based on this structure model. Predicted functions were closely related to the GO terms of the protein complex (GO:0043234) and the phosphatidylinositol 3-kinase (PI3K) complex (GO: 0005942) in the cellular component category with high confidence scores of 0.89–1.00 (Figure 4b; Supplementary Table S1).

## 4. Discussion

Since the first discovery of NAP50 in 2009, it has remained a mysterious protein in terms of its function. NAP50 is abundantly expressed in the dinoflagellate plastid [8], indicating that it may play important biological roles in plastid function. This study investigated the genetic structure, phylogenetic relationships, distribution across lineages, and structure-based functional predictions of NAP50, which contribute to our insights into the function of the NAP50 protein in dinoflagellates.

NAP50 is a typical nuclear-encoded plastid protein in dinoflagellates. Abundant base substitutions and no intron were found in the NAP50 gene of *A. affine* in this study. Amino acid substitutions of the NAP50 protein have also been reported in the *A. tamarense* complex [12]. The characteristics of the NAP50 gene are consistent with the general features of dinoflagellate nuclear genes; that is, highly expressed genes are often organized in tandem repeats that encode protein isoforms and exhibit frequent synonymous substitutions while lacking introns [13]. These genetic features suggest that the regulation of NAP50 expression may be primarily post-transcriptional, which is consistent with previous studies [10].

Although some dinoflagellate NAP50 proteins were annotated as TDP2, NAP50 exhibited notable differences in conserved sites to typical TDP2 proteins (Figure 1b). NAP50 and TDP2 proteins were both identified in the genomes of some dinoflagellate species, while others lacked the TDP2 or NAP50 proteins. Phylogenetic analysis showed that the dinoflagellate NAP50 proteins clustered into a separate branch with TDP2 and were more closely related to the TDP family of proteins in ciliates (Figure 2). These findings suggest that NAP50 could represent a unique member of the TDP family of proteins in some dinoflagellate lineages. It has been reported that TDP proteins (TDP1 and TDP2) repair DNA damage induced by aberrant topoisomerase activity by hydrolyzing the covalent bond between topoisomerases and DNA, thereby preventing the accumulation of single-strand breaks and double-strand breaks [14]. However, there are few sequence or structural similarities between the TDP1 and TDP2 proteins, despite the complementary functions of the two proteins [15]. A similar situation may exist between the NAP50 and TDP2 proteins, accounting for the differences in conserved amino acids between NAP50 and typical TDP2 proteins. TDP2 is undoubtedly the protein with the highest sequence similarity to NAP50 in the known databases. Considering the abundance of dark proteins encoded by dinoflagellate genomes, NAP50 proteins may not be an isolated case; rather, they are unique gene products that have evolved in dinoflagellates to adapt to their environments. Although the possibility of incomplete genomic data cannot be ruled out, the lineage-specific evolutionary traits could explain why either TDP2 or NAP50 was detected in certain dinoflagellate species analyzed in this study.

The distribution of NAP50 across dinoflagellate lineages was investigated using both bioinformatics analysis and Western blotting. Proteins from the dinoflagellate order Suessiales were found to share over 50% similarity with the NAP50 sequence (Figure 1a), which suggests that NAP50 is not limited to the genus *Alexandrium* and is more conserved within dinoflagellates than previously thought [16]. Western blotting was also used to validate the prevalence of NAP50 in dinoflagellates, particularly for species that lacked genomic data. Western blotting results showed that NAP50 was widely distributed in dinoflagellates, including species from the genera *Alexandrium*, *Amphidinium*, *Gymnodinium*, and *Prorocentrum* (Figure 3). Two bands with very similar molecular weights near 50 kDa were found in *P. hoffmannianum*, which could be the isoforms of NAP50 proteins [10]. The NAP50 protein was not detected in *S. acuminata* or *K. mikimotoi*, probably due to the following reasons. First, substantial divergence in the dark protein sequences has been observed across dinoflagellate lineages [7], which may limit the effectiveness of NAP50 antibodies developed from the *A. affine* protein sequence in detecting NAP50 from distantly related species. Second, this study revealed the presence of TDP2, but not NAP50, in specific dinoflagellate species through bioinformatics analysis. TDP1 and TDP2 have complementary functions in DNA repair [15]; therefore, it is possible that these species may utilize the canonical form of TDP2 rather than NAP50 for repairing DNA damage. Nevertheless, these hypotheses need to be confirmed by further research on the identification of additional NAP50 sequences in various dinoflagellate species, as well as experimental evidence of the role of NAP50 in DNA repair.

Nowadays, deep learning algorithms have significantly contributed to the advances in the prediction of protein three-dimensional structure from amino acid sequences [17]. In this study, the NAP50 protein structure was successfully folded with D-I-TASSER, as evidenced by a high eTM score (0.81). The structure-based function predictions of NAP50 were closely related to the GO terms “protein complex” and “PI3K complex” (Figure 4). It has been reported that the members of the phosphatidylinositol 3-kinase-related kinase (PIKK) family play crucial roles in the DNA damage response signaling pathway [18]. PIKKs promote TDP1 activity and function in synergism with TDPs to repair DNA damage [19,20]. TDPs have been shown to repair trapped topoisomerases and promote gene transcription in the mitochondria of human cells [21,22]. It is well known that photosynthesis in plastids generates reactive oxygen species, leading to photo-oxidative stress if not efficiently scavenged. Many plastid-derived genes have been transferred to the nucleus in peridinin-containing dinoflagellates [23], which is thought to minimize oxidative stress [24]. NAP50 is encoded by the nucleus but is predominantly expressed in the plastid [10], suggesting its important role in repairing DNA damage and maintaining the normal functions of the plastid in dinoflagellates. In the present study, NAP50 was expressed in many peridinin-containing dinoflagellate species (except *S. acuminata*) but was absent in *K. mikimotoi* possessing a haptophyte-derived plastid (Figure 3). Whether the presence of NAP50 is correlated with the plastid type needs further investigation on a wider spectrum of dinoflagellate plastid lineages. The present study suggests that the dinoflagellate NAP50 protein might be a member of the TDP family, potentially playing a role in DNA repair, and even contributing to plastid gene transcription in certain dinoflagellate plastids. More genomic information and related functional experimental validation are needed to enhance our understanding of the abundant dark proteins and their functions in dinoflagellates.

## 5. Conclusions

The NAP50 protein of *A. affine* is abundantly expressed in dinoflagellate plastids, but its function remains poorly characterized. The present study found that NAP50 is widely distributed across different dinoflagellate lineages. Phylogenetic analyses revealed that NAP50 could represent a novel and unique member of the TDP family of proteins that evolved in some dinoflagellate lineages. Structure-based functional predictions suggested that NAP50 may play a role in repairing DNA damage and contributing to the transcription of plastid genes in dinoflagellate species. This study not only sheds light on the dark protein (NAP50) that is abundant in the plastid with its distinct genetic sequence and its potential contributions in maintaining plastid functions, but it also serves as an example of adaptive evolution in dinoflagellates, providing a foundation for future research. However, these suggested functions of NAP50 and their significance in dinoflagellate biology need further exploration. More omics data on dinoflagellates and functional experiments in future would help us to gain a deeper understanding of these aspects.

## Data Availability

The original contributions presented in the study are included in the Supplementary Material; further inquiries can be directed to the corresponding author.

## Supplementary Materials

The following supporting information can be downloaded at https://www.mdpi.com/article/10.3390/microorganisms12112286/s1: Figure S1: Alignment of the NAP50 sequences from PCR and database; Figure S2: Blast results of NAP50 protein in the SAGER database; Figure S3: Original image of the Western blots. Table S1. GO terms of the protein complex (GO:0043234) and the phosphatidylinositol 3-kinase (PI3K) complex (GO: 0005942).

## References

[1] Taylor F.J.R., Hoppenrath M., Saldarriaga J.F. (2007). Dinoflagellate diversity and distribution. Biodivers. Conserv..

[2] Dagenais-Bellefeuille S., Morse D. (2013). Putting the N in dinoflagellates. Front. Microbiol..

[3] LaJeunesse T.C., Parkinson J.E., Gabrielson P.W., Jeong H.J., Reimer J.D., Voolstra C.R., Santos S.R. (2018). Systematic revision of Symbiodiniaceae highlights the antiquity and diversity of coral endosymbionts. Curr. Biol..

[4] Grattan L.M., Holobaugh S., Morris J.G. (2016). Harmful algal blooms and public health. Harmful Algae.

[5] LaJeunesse T.C., Lambert G., Andersen R.A., Coffroth M.A., Galbraith D.W. (2005). *Symbiodinium* (Pyrrhophyta) genome sizes (DNA content) are smallest among dinoflagellates1. J. Phycol..

[6] Wisecaver J.H., Hackett J.D. (2011). Dinoflagellate genome evolution. Annu. Rev. Microbiol..

[7] Gonzalez-Pech R.A., Stephens T.G., Chen Y., Mohamed A.R., Cheng Y., Shah S., Dougan K.E., Fortuin M.D.A., Lagorce R., Burt D.W. (2021). Comparison of 15 dinoflagellate genomes reveals extensive sequence and structural divergence in family Symbiodiniaceae and genus *Symbiodinium*. BMC Biol..

[8] Stephens T.G., Ragan M.A., Bhattacharya D., Chan C.X. (2018). Core genes in diverse dinoflagellate lineages include a wealth of conserved dark genes with unknown functions. Sci. Rep..

[9] Waller R.F., Kořený L. (2017). Plastid complexity in dinoflagellates: A picture of gains, losses, replacements and revisions. Adv. Bot. Res..

[10] Lee F.W., Morse D., Lo S.C. (2009). Identification of two plastid proteins in the dinoflagellate *Alexandrium affine* that are substantially down-regulated by nitrogen-depletion. J. Proteome Res..

[11] Zheng W., Wuyun Q., Freddolino P.L., Zhang Y. (2023). Integrating deep learning, threading alignments, and a multi-MSA strategy for high-quality protein monomer and complex structure prediction in CASP15. Proteins.

[12] Li C., Zhang Y., Xie Z.X., He Z.P., Lin L., Wang D.Z. (2013). Quantitative proteomic analysis reveals evolutionary divergence and species-specific peptides in the *Alexandrium tamarense* complex (Dinophyceae). J. Proteom..

[13] Bachvaroff T.R., Place A.R. (2008). From stop to start: Tandem gene arrangement, copy number and trans-splicing sites in the dinoflagellate *Amphidinium carterae*. PLoS ONE.

[14] Pommier Y., Huang S.Y., Gao R., Das B.B., Murai J., Marchand C. (2014). Tyrosyl-DNA-phosphodiesterases (TDP1 and TDP2). DNA Repair.

[15] Kawale A.S., Povirk L.F. (2018). Tyrosyl-DNA phosphodiesterases: Rescuing the genome from the risks of relaxation. Nucleic Acids Res..

[16] Lee F.W.F. (2008). Proteomic Study of Harmful Algal Blooming Causative Agents: Nitrogen-Induced Growth and Identification of Dinoflagellates. Ph.D. Dissertation.

[17] Peng Z., Wang W., Han R., Zhang F., Yang J. (2022). Protein structure prediction in the deep learning era. Curr. Opin. Struct. Biol..

[18] Lovejoy C.A., Cortez D. (2009). Common mechanisms of PIKK regulation. DNA Repair.

[19] Das B.B., Antony S., Gupta S., Dexheimer T.S., Redon C.E., Garfield S., Shiloh Y., Pommier Y. (2009). Optimal function of the DNA repair enzyme TDP1 requires its phosphorylation by ATM and/or DNA-PK. EMBO J..

[20] Katyal S., Lee Y., Nitiss K.C., Downing S.M., Li Y., Shimada M., Zhao J., Russell H.R., Petrini J.H., Nitiss J.L. (2014). Aberrant topoisomerase-1 DNA lesions are pathogenic in neurodegenerative genome instability syndromes. Nat. Neurosci..

[21] Chiang S.C., Meagher M., Kassouf N., Hafezparast M., McKinnon P.J., Haywood R., El-Khamisy S.F. (2017). Mitochondrial protein-linked DNA breaks perturb mitochondrial gene transcription and trigger free radical-induced DNA damage. Sci. Adv..

[22] Huang S.N., Dalla Rosa I., Michaels S.A., Tulumello D.V., Agama K., Khiati S., Jean S.R., Baechler S.A., Factor V.M., Varma S. (2018). Mitochondrial tyrosyl-DNA phosphodiesterase 2 and its TDP2(S) short isoform. EMBO Rep..

[23] Howe C.J., Nisbet R.E., Barbrook A.C. (2008). The remarkable chloroplast genome of dinoflagellates. J. Exp. Bot..

[24] Dorrell R.G., Howe C.J. (2012). What makes a chloroplast? Reconstructing the establishment of photosynthetic symbioses. J. Cell. Sci..

